# Psychiatry’s response to the climate change emergency

**DOI:** 10.1192/j.eurpsy.2023.171

**Published:** 2023-07-19

**Authors:** V. Pereira-Sanchez

**Affiliations:** Child and Adolescent Psychiatry, New York University Grossman School of Medicine, New York, United States

## Abstract

**Abstract:**

Dr. Pereira-Sanchez will discuss how the climate change emergency apeals psychiatrists and demands both personal and organized responses. Such responses are in the domains of awareness, research, education, and action. Dr. Pereira-Sanchez will present specific examples from his collaborative work at the World Network of Psychiatric Trainees, where a global forum about the topic for trainees was organized, and the World Psychiatric Association, where he assists the coordination of a Tri-Sectional initiative on the topic.

**Disclosure of Interest:**

None Declared

